# Engineering of Antimicrobial Surfaces by Using Temporin Analogs to Tune the Biocidal/antiadhesive Effect

**DOI:** 10.3390/molecules24040814

**Published:** 2019-02-24

**Authors:** Pierre-Carl Oger, Christophe Piesse, Ali Ladram, Vincent Humblot

**Affiliations:** 1 Sorbonne Université, CNRS, Laboratoire de Réactivité de Surface, LRS UMR CNRS 7197, F-75252 Paris, France; pierre-carl.oger@hotmail.com; 2 Sorbonne Université, CNRS, Institut de Biologie Paris-Seine, IBPS, F-75252 Paris, France; christophe.piesse@sorbonne-universite.fr (C.P.); ali.ladram@sorbonne-universite.fr (A.L.)

**Keywords:** temporin-SHa, SHa analogs, antimicrobial peptides, gold surface functionalization, antibacterial/antiadhesive activity

## Abstract

Proliferation of resistant bacteria on biomaterials is a major problem leading to nosocomial infections. Due to their broad-spectrum activity and their ability to disrupt bacterial membranes through a rapid membranolytic mechanism, antimicrobial peptides (AMPs) are less susceptible to the development of bacterial resistance and therefore represent good candidates for surface coating strategies to prevent biofilm formation. In this study, we report on the covalent immobilization of temporin-SHa, a small hydrophobic and low cationic antimicrobial peptide exhibiting broad-spectrum activity, and (SHa) analogs on modified gold surfaces. Several analogs derived from SHa with either a carboxamidated ([K^3^]SHa, d-[K^3^]SHa) or a carboxylated C-terminus ([K^3^]SHa-COOH) were used to achieve peptide grafting on gold surfaces modified by a thiolated self-assembled monolayer (SAM). Surface functionalization was characterized by polarization modulation infrared reflection absorption spectroscopy (PM-RAIRS) and X-ray photoemission spectroscopy (XPS). The antibacterial properties of the temporin-functionalized surfaces were tested against the Gram-positive *Listeria ivanovii*. Direct visualization of the peptide effects on the bacterial membrane was investigated by scanning electron microscopy equipped with a field emission gun (SEM-FEG). All active temporin analogs were successfully grafted and display significant antibacterial activity (from 80 to 90% killing efficiency) in addition to a 2-fold decrease of bacterial adhesion when all d-SHa analogs were used.

## 1. Introduction

More than 70 years ago, the scientific community described for the first time bacterial contaminations on surfaces that later would be named biofilms [1]. A biofilm is a more or less complex multicellular community, often composed of symbiotic microorganisms (such as bacteria, fungi and algae) adhering to a surface or together as aggregates, and characterized by the secretion of a protective and adhesive matrix. Consequently, bacteria can be found in two different states when encountered in natural environment: planktonic, i.e., free in media, and sessile, anchored or attached to a surface [2]. Biofilms generally form in water or in an aqueous medium [3]. The biofilm matrix usually encapsulates and protects the embedded bacteria, thus conferring high resistance to surrounding stresses [4]. The biofilm is a potentially normal step of the life cycle of most bacteria, displaying cooperative behavior and producing differentiated phenotypes that lead to specific functions, sometimes in response to stress [5].

It is now established that the development of a biofilm occurs in several steps. It is initiated by the formation of a primary film, which results from the adsorption or the absorption of organic macromolecules (such as proteins, polysaccharides and lipids) and the liquid present in inorganic phase. This phenomenon leads to the change of physical and chemical properties of the surfaces and promotes bacterial adhesion. Nowadays, biofilms are still causing acute problems in a broad range of fields, from the biomedical to the environmental and food industries [6,7]. Two routes are commonly investigated in order to address the issue of biofilm development, one aiming at preventing the adsorption/adhesion of biomolecules/bacterial cells and the other at inhibiting/killing adhered microorganisms. Recent work has been done in this direction with the use of hydrophilic polymers (i.e., poly ethylene glycol or ulvan) or surface-bound enzymes/antibacterial peptides [8,9,10]. These chemical treatments significantly reduce the adhesion and growth of microorganisms but only for limited periods of time.

Several biocidal agents, such as antibiotics [11], quaternary ammonium [12,13], titanium oxide [14] or metallic particles (silver or tin) [15,16], to cite a few, have been immobilized on surfaces. However, their use is more and more forbidden due to their toxicity. Therefore, the use of natural antimicrobial peptides (AMPs), which are produced by mammals, insects or even plant micro-organisms [17], appear as an alternative to conventional antibiotics. AMPs present several advantages: efficiency at low concentration, broad-spectrum activity towards several micro- organisms or even more importantly, less susceptibility to bacterial resistance compared to conventional antibiotics [18,19]. We have previously adsorbed AMPs, such as magainin, gramicidin or nisin, on several surfaces and showed that they retained their antimicrobial activity upon grafting [9,10,20]. We have also investigated the functionalization of gold surfaces with another family of short AMPs, the temporins, and more specifically with temporin-SHa (SHa) [21]. Results have shown that depending on the anchoring mode of SHa, the killing activity of the grafted surface was different. SHa is a 13-residue AMP isolated from the North African ranid frog *Pelophylax saharicus* with broad-spectrum activity against Gram-positive and Gram-negative bacteria, yeasts and parasites [22]. This peptide adopts an amphipathic α-helical structure in apolar media or in membrane mimetic environments [23].

**Figure 1:**
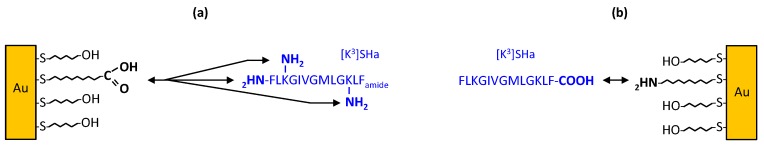
Different grafting strategies of SHa analogs. (**a**) Grafting of C-terminal α-carboxamidated SHa analogs on carboxylic acid-terminated MUA SAMs. (**b**) Grafting of free C-terminal carboxylate SHa analogs on amine-terminated MUAM SAMs. Reactive functions of the MUA/MUAM SAM and of [K^3^]SHa are indicated in bold.

The present study aimed at analyzing the antimicrobial activity of different SHa analogs after grafting on gold surfaces, and determining the parameters influencing such activity, based on the complex interplay between activity and physicochemical properties (length, secondary structure, net positive charge, hydrophobicity, helicity and amphipathicity) [24,25,26]. Because recently, structure-activity relationship studies allowed us to identify [K^3^]SHa as a highly potent analog compared to the parent peptide SHa [27], we choose this analog in our study. [K^3^]SHa has a Lys residue in position 3 instead of a Ser residue, thereby increasing the net positive charge of SHa to a value of +3. We also synthesized and used the D-[K^3^]SHa enantiomer (all-D α-C configuration) to analyze possible conformational effects of bound [K^3^]SHa. The analog [A^2,6,9^, K^3^]SHa, where Leu^2,9^ and Val^6^ residues of [K^3^]SHa were replaced with Ala to reduce the hydrophobicity of the apolar face of the amphipathic α-helix, was demonstrated to be inactive in solution [27] and was therefore chosen as a negative control in our study. Adsorption of temporins (C-terminal carboxamidated and carboxylated) on gold surfaces was achieved by using different grafting strategies depending on the chosen peptide, either via amine- or carboxylic acid-terminated self-assembled monolayers (SAM, Figure 1). All functionalized surfaces were thus analyzed by means of infrared spectroscopy (PM-RAIRS) and photoemission spectroscopy (XPS). Antimicrobial activity of the functionalized surfaces was assessed against the Gram-positive bacteria *Listeria ivanovii* after determining in solution the antibacterial activity of the free peptides.

## 2. Results and Discussion

### 2.1. Antibacterial Activity of the Free Temporins in Solution

We first investigated the antimicrobial activity of temporin analogs by determining minimal inhibitory concentrations (MICs) and minimal bactericidal concentrations (MBCs) against a Gram-positive bacterial strain, *Listeria ivanovii* (Li4pVS2). The results obtained in this study are presented in Table 1 for all temporin analogs used in this study and are compared to the values of the parent SHa peptide. At first, these values confirmed the high potency of both L- and D-[K^3^]SHa enantiomers, with values of 1.56 and 3.12 µM for MIC and MBC, respectively, compared to SHa analog (MIC = MBC = 6 µM). These results confirmed those obtained in our previous study indicating that increasing the net positive charge of SHa to a value of +3 leads to a more efficient analog [27]. Both [K^3^]SHa enantiomers were equipotent towards *L. ivanovii*, with an increase by a factor 4 of the inhibition activity and a factor 2 for the bactericidal activity. However, when looking at the [K^3^]SHa-COOH analog, the MIC and MBC values increased up to 64 and 128 µM, respectively. This could be due to the lower net positive charge of [K^3^]SHa-COOH (+ 2) compared to C-terminal caboxamidated [K^3^]SHa (+3). In addition, one should note that the ratio MBC/MIC is always below 4, thus indicating bactericidal activity of the temporin analogs rather than bacteriostatic activity [28]. As previously shown [27], the two alanine-substituted analogs, [A^2,6,9^]SHa and [A^2,6,9^, K^3^]SHa are inactive against *L. ivanovii* strain (MIC and MBC > 200 μM). [A^2,6,9^, K^3^]SHa-COOH is also virtually inactive, with MIC and MBC corresponding to 128 µM and 256 μM, respectively. These three peptides were then used as negative controls.

### 2.2. Surface Characterization

**Figure 2:**
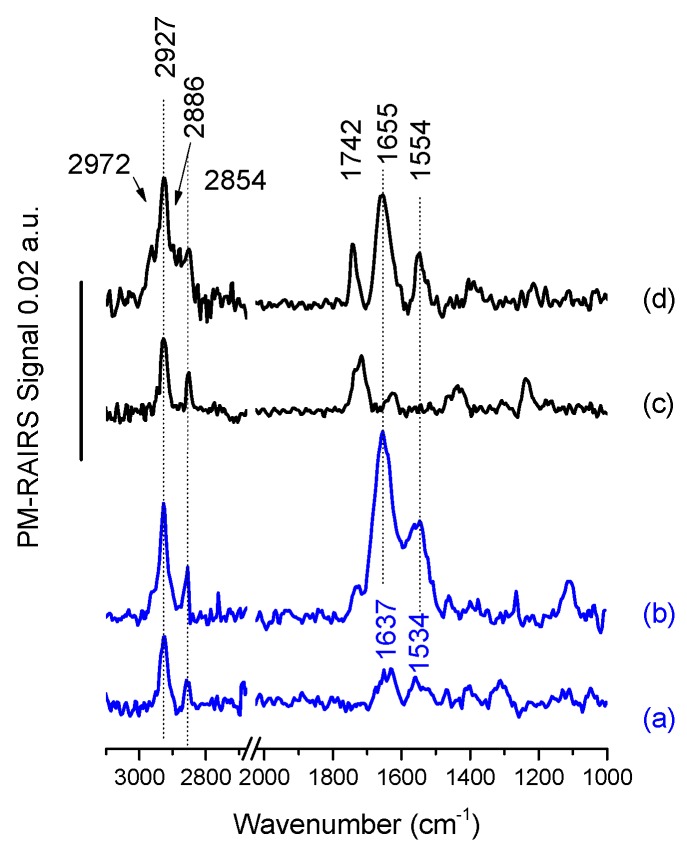
PM-RAIRS spectra of gold surfaces functionalized with (**a**) MUAM, (**b**) MUAM-[K^3^]SHa-COOH, (**c**) MUA and (**d**) MUA-[K^3^]SHa.

The PM-RAIRS spectra of gold surfaces modified by carboxylic acid and amine thiols, and the corresponding temporins are presented in Figure 2.

Considering first the grafting of the amine thiol, 11-amino-1-undecanethiol or MUAM (Figure 2a), three main vibration bands are observed. One positioned at 1360 cm^−1^ can be attributed to the ωCH_2_ scissoring vibration of the alkyl backbone of the MUAM and a second set is positioned at 1637 and 1534 cm^−1^ assigned to the δNH_3_^+^ and to the asymmetric δNH_2_, respectively. Finally, the symmetric and antisymmetric stretching vibrations of the CH_2_ groups are observed at 2854 and 2927 cm^−1^, respectively [20,29]. Figure 2b shows the appearance of several new and intense IR bands after temporin [K^3^]SHa-COOH adsorption. Two strong features corresponding to the amide I and amide II bands of the peptide backbone are visible at 1655 and 1554 cm^−1^. Another band at around 1720 cm^−1^ is also observed and assigned the stretching C=O vibration of some non-activated temporins probably attached to the surface with non-specific interaction despite the heavy rinsing protocols. All these IR features indicate the successful covalent binding of [K^3^]SHa-COOH on the top of a MUAM self-assembled monolayer (SAM).

Turning now to the carboxylic acid thiol SAM composed of 11-mercaptoundecanoic acid or MUA, Figure 2c shows the PM-RAIRS spectrum recorded after the functionalization of the gold surface with MUA. The IR spectrum is dominated by a strong C=O stretching band at 1722 cm^−1^, distinctive of carboxylic groups [10,30,31], accompanied with a smaller feature at 1244 cm^−1^ attributed to the OH deformation; both features are showing the presence of the acid-terminated thiol. Three weak additional features are also observable at ~1420 cm^−1^ and ~1620 cm^−1^, assigned to stretching vibrations of deprotonated COO^–^ groups, and a last peak at 1455 cm^−1^ likely including contributions from the scissor mode of CH_2_ groups. Finally, in the higher wavenumber region, two bands corresponding the CH_2_ stretching mode are observed at 2854 cm^−1^ and 2927 cm^−1^. The presence of these characteristic IR vibrations confirms the successful elaboration of the MUA SAM on the gold surface. After the activation step (see [10]) the gold surfaces were immersed during 3 h in the solutions containing the L- and D-analogs of [K^3^]SHa temporins. The corresponding PM-RAIRS spectrum for L-analog is presented in Figure 2d. As observed previously for the binding of [K^3^]SHa-COOH on MUAM SAM, the spectrum is now dominated by two intense IR features at 1655 and 1544 cm^−1^, corresponding again to the amide bands of the peptide backbone. One should also note the presence of a feature at 1742 cm^−1^ that is the C=O stretching signature of the activated ester band not involved in the creation of an amide bond. Finally, four bands corresponding to the stretching mode of alkyl groups (CH_2_ and CH_3_) are observed between 2972 and 2854 cm^−1^. Again, the presence of these IR features clearly indicates the successful binding of L-[K^3^]SHa on the carboxylic acid SAM. The data obtained with PM-RAIRS (data not shown) also confirmed the successful grafting of the other temporin analogs: D-[K^3^]SHa, L-[A^2,6,9^, K^3^]SHa and L-[A^2,6,9^, K^3^]SHa-COOH.

**Figure 3:**
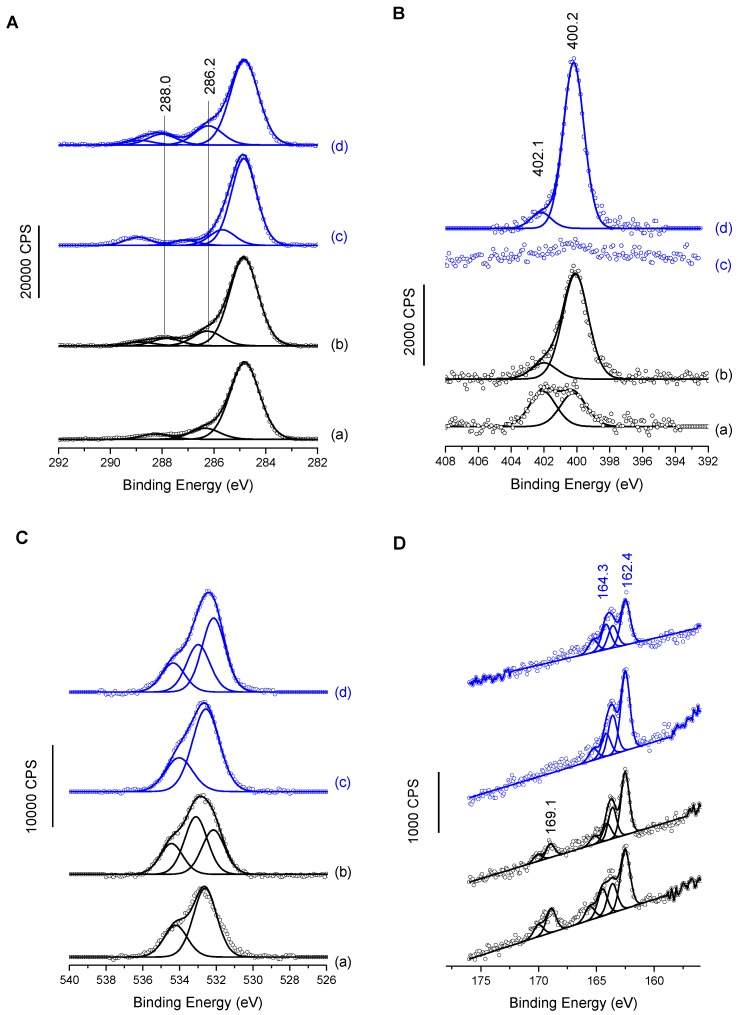
High resolution XPS spectra of gold surfaces functionalized with (a) MUAM, (b) MUAM-[K^3^]SHa-COOH, (c) MUA and (d) MUA-[K^3^]SHa for (**A**) the C1s region, (**B**) the N1s region, (**C**) the O1s region and (**D**) the S2p region.

XPS analyses after MUA/MUAM immobilization and temporin binding provided complementary information. The C1s, N1s, O1s and S2p are presented in Figure 3. C1s and N1s MUAM spectra of the Figure 3A(a),B(a) exhibit all components expected for adsorbed thiol amine on gold surface, especially the presence of protonated and deprotonated amine groups for N1s (contributions at 400.2 ± 0.1 and 402.1 ± 0.1 eV, respectively) and two main components for aliphatic carbon atoms, with one main peak at 285.0 eV, and a smaller one at 286.2 ± 0.1 eV, for carbon in a position of an heteroatom (i.e., C-S and C-N in the present case) [20]. MUA spectra in Figure 3A(c),B(c) also exhibit the usual features of the grafted carboxylated molecule, with no nitrogen contribution and for the carbon C1s region, the 2-identical low binding energy contributions and a third one at 289.0 ± 0.1 eV assigned to carboxylic acid groups [32]. O1s regions for both primers (MUA and MUAM) exhibit an oxygen signal best fitted with two components. Although it is expected in the case of MUA, Figure 3C(c), due to the presence of acidic head group, it is less expected in the case of MUAM, Figure 3C(a). However, when looking at the different atomic percentages for these surfaces, Table 2, MUAM surface exhibits only 13% of atomic oxygen compared to 21% in the case of MUA. This value is high for MUAM, but not so unusual when taking into account the purity of the organic product and also the fact that surface functionalization is carried out in the liquid phase. It is important to note that when temporin analogs are grafted onto these SAMs, the O1s signals now bear an enlarged peak with the appearance of a third contribution, Figure 3C(b,d), due to the presence of the peptides on the surfaces. Finally, the S2p region also shows interesting results, Figure 3D. First of all, for both surfaces coming from the MUAM primers (Figure 3D(a,b), one can see oxidized sulfur signal at high binding energies around 169.1 eV, mainly due to the presence of oxygen within the adlayers as observed previously. Turning to the low binding energy components at 164.3 and 162.4 eV present in all four spectra, these components are assigned to unbound and bound thiol-sulfur, respectively. Again, this phenomenon is not unusual when engineering thiolated self assembled monolayers despite extensive rinsing process, with only a small amount of unbound thiolated molecules. Finally, one can notice the relative decrease of the S2p signal intensity from MUAM (MUA) to MUAM [K^3^]SHa-COOH (MUA [K^3^]SHa) due to its higher attenuation by a thicker organic layer.

Surfaces grafted with temporins were also investigated by XPS (Figure 3A,B, c and d for both C1s and N1s regions). N1s high-resolution spectrum for MUAM [K^3^]SHa-COOH (Figure 3B(b)), shows an increase of the 400 eV contribution and a decrease of the 402 eV one. The former being now assigned to both NH_2_ amine group and NH group from the amide bond of the peptide. Concerning the C1s region, although the aliphatic carbon (285 ev) contribution shows only a small increase, an increase of the binding energy intensity of the α-carbon at 286.2 ± 0.1 eV (together with the C=O component at 288.0 ± 0.1 eV) clearly demonstrates the successful grafting of [K^3^]SHa-COOH on Au-MUAM.

Adsorption of temporin [K^3^]SHa on MUA modified gold surface is mainly observed by the appearance of a nitrogen peak, N1s, centered at 400.2 ± 0.1 eV, principally due to the amide functions of the peptide [33,34] (Figure 3B(d) with a smaller contribution at 402.1 ± 0.1 eV originating from protonated amine groups of the temporin lateral chains. Figure 3A(d) shows high-resolution XPS spectrum of the C1s core level peaks corresponding to Au-MUA-[K^3^]SHa, which was best fitted with four contributions at 284.9, 286.2, 288.0 and 289.0 ± 0.1 eV, corresponding respectively to the aliphatic carbon in C–C, C–H bonds, C in the alpha position, and finally C atoms involved in peptide bonds, O=C–N and acid groups (H-O–C=O) [33,35]. Again, these XPS data confirm the successful grafting of temporins on Au-MUA SAMs. Note that only [K^3^]SHa data are presented in Figure 3, but the same experiments have been carried out for the other temporin analogs with the equivalent results.

From these XPS data, one can also estimate the average thickness of the adlayer and then deduce the density of grafted temporin molecules at the surface. The details of the calculations can be found elsewhere [32,36,37] but the principle is briefly reminded below. First, by looking at the I_S2p_/I_Au4f_ ratio at the thiol-gold interface, the thickness of both MUA and MUAM thiols SAMs can be calculated (Table 3). Considering a homogeneous adlayer, these thicknesses are close to 11 Å, which is the usual thickness observed for C10 or C11 aliphatic thiol SAMs [31,38]. From these values, the surface density of each molecule can also be obtained. The values obtained are in very good agreement with what was observed in the literature, with a molecular surface density close to 3 thiol molecules/nm², keeping in mind that the maximum thiol density is 4 molecules/nm² [21,29,39,40]

Turning to the same calculations for the temporin-modified surfaces, Table 3, using the same formulae, gives the total thickness of the adlayer, to which the thiol thickness must be subtracted to obtain to thickness of the temporin layer alone, and hence the temporin molecular surface density. Thus, on average, the grafting efficiency is around 1/3 with about one temporin molecule grafted per three available thiols anchoring point, which is the average efficiency observed due to steric hindrance when adsorbing peptides, glycolipids or proteins on SAMs [29,32,41]. This value of ~1 active molecule per nm² is within the range usually observed in several systems. For instance, a small arginine branched tripeptide grafted on silicone average 0.2 peptide/nm² [42] while and α-helical peptide on gold rises up to 1 molecule/nm² [37].

### 2.3. Antibacterial Activity of Grafted Temporins

**Figure 4:**
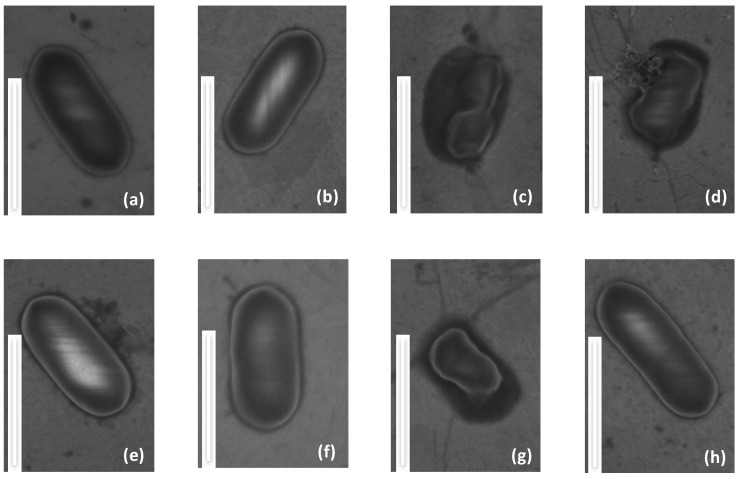
Structural morphology of the Gram-positive bacteria *Listeria ivanovii* in contact with grafted surfaces compared to bare gold. (**a**) Au, (**b**) MUA, (**c**) [K^3^]SHa, (**d**) d-[K^3^]SHa, (**e**) [A^2,6,9^, K^3^]SHa, (**f**) MUAM, (**g**) [K^3^]SHa-COOH and (**h**) [A^2,6,9^, K^3^]SHa-COOH. The scale bare represents 1 μm.

In order to evaluate the antimicrobial activity of the temporin-functionalized gold surfaces, we have combined scanning electron microscopy (SEM-FEG) with microbial viability tests, on both modified and clean gold surfaces. SEM-FEG is a fast screening technique to observe possible changes of the morphology of bacteria. To avoid possible artifacts due to intrinsic observation conditions of SEM, i.e., high vacuum, bacteria have been fixed with glutaraldehyde 2.5% in PBS. Bacteria are of about 1 μm in length and exhibit the expected elongated rod shape [43,44]. SEM-FEG images in Figure 4a,b,f show *L. ivanovii* deposited on temporin-free surfaces (Au, MUA and MUAM) where the plasma membrane of this Gram-positive bacteria appears as a white line surrounding the rod-shaped microorganism, indicating that contact with these surfaces does not alter the cell envelope. On the contrary, on the temporin-modified surfaces ([K^3^]SHa, D-[K^3^]SHa and [K^3^]SHa-COOH), bacteria do not have their native oval shape anymore, and the cell envelopes are damaged or squeezed (Figure 4c,g) and appear pierced, causing the leakage of the cytoplasmic content and the collapse of the bacteria (Figure 4d). These observations show direct evidence that the gold surface grafted with [K^3^]SHa analogs damages the plasma membrane of *L. ivanovii* in agreement with the antibacterial effect reported for temporins in solution [27,45,46] (Table 1). Finally, it is worth noting that, as expected from the MIC and MBC values obtained from in vitro assays, [A^2,6,9^]SHa analogs do not show any antibacterial activity towards *L. ivanovii* once grafted on a gold surface (Figure 4e,h).

**Figure 5:**
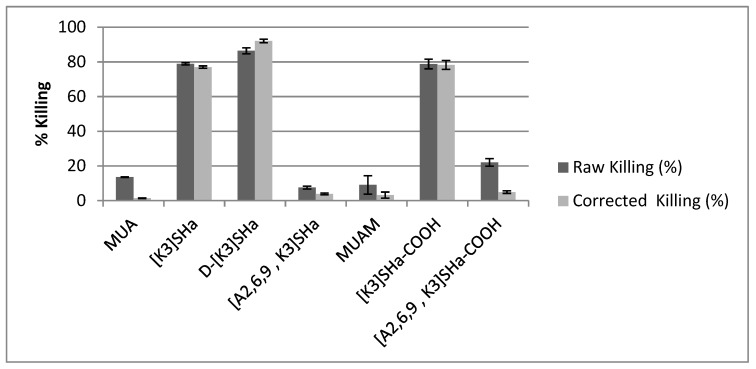
Killing efficiency towards *L. ivanovii* bacteria compared to gold bare substrate for MUA and MUAM, to MUA for [K^3^]SHa, d-[K^3^]SHa, [A^2,6,9^, K^3^]SHa, and to MUAM for [K^3^]SHa-COOH and [A^2,6,9^, K^3^]SHa-COOH, respectively. The raw data (dark bars) are compared to the data corrected by the adhesion factor (light bars). The killing of X compared to its control is calculated using %Killing_X_ = [(CFU_CONTROL_ − CFU_X_)/CFU_CONTROL_)] × 100. Error bars indicate standard deviation from three independent CFU counts.

However, electron microscopy does not constitute a direct proof of bacteria viability or death, and most importantly does not provide a quantification of the potential antibacterial efficiency of the modified surfaces. Conversely, bacteria were also cultivated on agar plate after having been in contact with the modified surfaces in order to count the actual number of viable bacterial colonies compared to bare gold surfaces. Results showing the %killing of the different grafted surfaces are summarized in Table 3, and are also presented in Figure 5 as dark bars under the label “raw killing”. At first glance, it is clear that there are three modified surfaces that exhibit high bactericidal efficiency with killing percentage around 80%, namely L- and D-[K^3^]SHa as well as [K^3^]SHa-COOH, which confirms what was observed by SEM-FEG. It is also worth noting that both anchoring primers (MUA and MUAM) show little bactericidal activity, around 13% and 9% respectively. Although, the surface modified with [A^2,6,9^, K^3^]SHa reveals only 7% of killing, as expected from the MIC/MBC values presented in Table 1, it is more unexpected to see the [A^2,6,9^, K^3^]SHa-COOH modified surface displaying a killing activity superior to 20% (Table 4).

**Figure 6:**
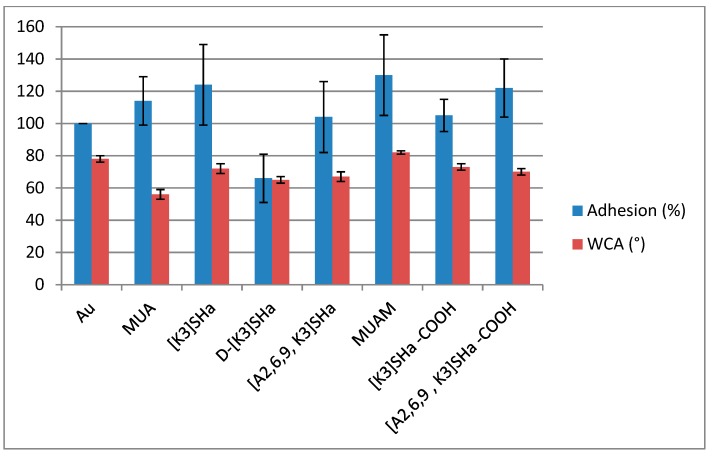
*L. ivanovii* bacterial adhesion (%) compared to gold bare substrate (100%), left bars. Water contact angle, WCA (°) measured on Au and modified surfaces, right bars.

Nevertheless, killing activities were different for our analogs, and we wanted to investigate what could be the cause of such drastic differences, especially for [A^2,6,9^] analogs. Thus we first investigated the hydrophobic character of the grafted surface by measuring sessile drop water contact angles (WCA) on all modified surfaces, compared to the bare gold surface. Results presented in Table 4 and Figure 6 do not show real differences for temporin analogs with WCA close to 70°, suggesting that the wettability of the surface did not influence the killing activity of the analogs. However, the net charge of the peptide could influence the molecular recognition between a given peptide and the bacteria membranes, lowering or increasing the affinity of the bacteria for the surface. This hypothesis can be verified by looking at the adhesion factor of a bacteria solution towards the modified surface.

Bacterial adhesion was then measured for all surfaces and normalized to the adhesion on bare gold (Table 4 and Figure 6). First, both primers (acid and amino thiols) showed an increase in adhesion compared to the gold reference, which in these two cases can be correlated with the wettability (72° for MUA and 82° for MUAM) and the charges (−1 for MUA and +1 for MUAM) of the surfaces with respect to the bacterial solution. However when looking at the adhesion for L-temporin analogs, values are ranging from 104% to 124%. These variations cannot really be explained by looking at the different parameters of each analog. For instance, there was a 20% decrease of the adhesion between [K^3^]SHa and [A^2,6,9^, K^3^]SHa for the same net charge of the peptide (+3) and the same surface density (~1 pept/nm²) and a similar wettability with WCA of ~70°. The tendency was the opposite when looking at the SHa-COOH analogs with an increase this time of around 20% from [K^3^]SHa-COOH to [A^2,6,9^, K^3^]SHa-COOH analogs although very similar WCA were again observed. However, one striking result was the decrease of the bacterial adhesion by more than 50% obtained for the all-D enantiomer D-[K^3^]SHa compared to the “natural” enantiomer [K^3^]SHa. This last result was clearly linked to the molecular interaction between bacteria and the temporin-modified gold surface containing two different L/D configurations, showing that the adsorption/adhesion processes of bacteria are somehow linked to molecular recognition and in this case to chiral recognition.

Finally, in order to properly compare the antibacterial activity of all analogs of temporin-SHa grafted on gold surface, the raw killing values must be corrected by the adhesion values presented above (see also Table 4). Thus, Figure 5 compares the raw killing values to the corrected ones. The correction does not influence too much the values of the three active analogs, [K^3^]SHa, [K^3^]SHa-COOH and D-[K^3^]SHa, with only a small increase for the latter from 86% to 92% killing efficiency. One can also note that the orientation of the peptide with respect to the surface within the adlayer does not influence the antibacterial activity, because a random adsorption mode obtained with [K^3^]SHa ends up with the same killing efficiency than a forced adsorption mode via the carboxylated C-terminal end ([K^3^]SHa-COOH, 78% of killing). However, for the inactive [A^2,6,9^, K^3^] analogs in solution, their activity upon grafting is now below 5% of killing, similar to the anchoring thiol primers.

## 3. Materials and Methods

### 3.1. Reagents

11-Mercaptoundecanoïc acid (MUA), 11-amino-1-undecanethiol (MUAM), N-hydroxy-succinimide (NHS), 1-(3-(dimethylamino)propyl)-N-ethylcarbodiimide hydrochloride (EDC), formaldehyde, dimethyl sulfoxide, sodium chloride (NaCl) and phosphate buffered saline (PBS) were obtained from Sigma-Aldrich (Saint Quentin Fallavier, France). All solvents were reagent-grade and were used without any further purification. Ultrapure water was obtained from a Milli-Q system (Millipore, resistivity >18 MΩ cm^−1^) from EMD Millipore Corp. (Billerica, MA, USA). Glass substrates (11 mm × 11 mm), coated successively with a 50 Å thick layer of chromium and a 200 nm thick layer of gold, were purchased from Arrandee (Werther, Germany). Temporins solutions were prepared in milli Q water solutions with phosphate buffer salts (PBS leading to a pH of 7.4). EDC/NHS solutions were prepared in milli Q water solutions with no further buffer.

### 3.2. Peptide Synthesis and Purification

Temporin-SHa (SHa, FLSGIVGMLGKLF_amide_), [K^3^]temporin-SHa ([K^3^]SHa, FLKGIVGMLGKLF_amide_), d-[K^3^]temporin-SHa (d-[K^3^]SHa, FLKGIVGMLGKLF_amide_), [A^2,6,9^]temporin-SHa ([A^2,6,9^]SHa, FASGIAGMAGKLF_amide_), [A^2,6,9^, K^3^]temporin-SHa ([A^2,6,9^, K^3^]SHa, FAKGIAGMAGKLF_amide_), [K^3^]temporin-SHa-COOH ([K^3^]SHa-COOH, FLKGIVGMLGKLF_COOH_), [A^2,6,9^, K^3^]temporin-SHa-COOH ([A^2,6,9^, K^3^]SHa-COOH, FAKGIAGMAGKLF_COOH_) were synthesized using solid-phase FastMoc chemistry on an Applied Biosystems 433A automated peptide synthesizer (Peptide Synthesis Platform, IBPS, Sorbonne University, Paris, France) and then purified by semi-preparative reversed-phase high-performance liquid chromatography (RP-HPLC), as previously described [21]. C-terminal α-carboxamidated peptides were prepared on a Rink Amide MBHA PS resin (Iris Biotech GmBH, Marktredwitz, Germany), whereas carboxylated peptides were prepared on a Fmoc-Phe-Wang resin LL (Novabiochem, Läufelfingen, Switzerland). Purity of the synthetic temporins was assessed by analytical RP-HPLC using an Aeris PEPTIDE column (XB-C18, 3.6 µm, 4.6 × 250 mm, Phenomenex, Le Pecq, France) eluted at 0.75 mL/min by a 20–70% linear gradient of acetonitrile (1% ACN/min) in 0.1% TFA/water, followed by MALDI-TOF mass spectrometry analysis (Mass Spectrometry and Proteomics Platform, IBPS, Sorbonne University, Paris, France). [M + H]^+^ theoretical (M_th_, using Peptide Mass Calculator v3.2 http://rna.rega.kuleuven.be/masspec/ pepcalc.htm) and experimental (M_exp_) molecular mass of synthetic peptides: SHa: M_th_ = 1380.81, M_exp_ = 1381,14; [K^3^]SHa: M_th_ = 1421.87, M_exp_ = 1421.84; d-[K^3^]SHa: M_th_ = 1421.87, M_exp_ = 1421.91; [K^3^]SHa-COOH: M_th_ = 1422.85, M_exp_ = 1422.86; [A^2,6,9^]SHa: M_th_ = 1268.55, M_exp_ = 1268.75; [A^2,6,9^, K^3^]SHa: M_th_ = 1309.64, M_exp_ = 1309.76; [A^2,6,9^, K^3^]SHa-COOH: M_th_ = 1310.64, M_exp_ = 1310.71.

### 3.3. Surface Preparation

The gold substrates (i.e., gold-coated surfaces) are prepared following a standard protocol [8]. before functionalization, the gold-coated substrates (Au) are annealed in a butane flame to obtain a crystal reconstruction of the first atomic layers; a UV-ozone cleaning procedure during 15 minis then applied prior to several ultrapure water and absolute ethanol rinsing for period of 10 min each.

For the grafting of [K^3^]SHa-COOH, first, the substrates were immersed in an ethanol solution of 10 mM MUAM (surfaces or substrates referred as MUAM) (each gold substrate is immersed separately in small Petri dish containing 5 mL of grafting solution). After 3 h, the substrates were sonicated in ultrapure ethanol to desorb the non-grafted molecules and thoroughly rinsed in ethanol, then in ultrapure water before being dried under a gentle dry N_2_ flow. Next, to promote the grafting of the C-terminally carboxylated temporins on the thiol-amine, the carboxylic group of [K^3^]SHa-COOH (20 mg·L^−1^) was activated by succinimide ester using a mixture of 1-ethyl-3-(3-dimethylaminopropyl)-carbodiimide (EDC, 77 mg·L^−1^) and N-hydroxysuccinimide (NHS, 23 mg·L^−1^) in water. After 1 h under stirring, the MUAM modified gold substrates were immersed for 3 h in this solution. Successive rinsing in ultrapure water and ethanol were performed to remove non-covalently grafted reactants before drying under dry N_2_ flow, the surfaces or substrates are referred as [K^3^]SHa-COOH).

Concerning the grafting of C-terminal carboxamidated temporins, first the gold substrates were immersed in a 10 mM solution of MUA in 10 mL of absolute ethanol for 3 h, in order to insure an optimal homogeneity of the adlayer, thoroughly rinsed in ethanol and MilliQ water and dried under a gentle nitrogen flow. The substrates (referred as MUA) were treated with a solution of 20 mM NHS and 10 mM EDC in ultrapure water for 90 min, rinsed in MilliQ water and dried under a dry N_2_ flow.

Then, immobilization of L- and D-[K^3^]SHa (20 mg·L^−1^) on MUA-functionalized gold surfaces was carried out by depositing the MUA modified gold substrates at room temperature for 3 h in the solution of temporins. After the immobilization step, the surfaces were vigorously rinsed in ultrapure water with agitation, dried under a dry N_2_ flow; the surfaces or substrates are referred as [K^3^]SHa. All grafting steps were carried out at room temperature except otherwise stated. All samples were characterized by PM-RAIRS and XPS after each step of functionalization.

### 3.4. Polarized Modulated Reflection Absorption Infrared Spectroscopy (PM-RAIRS)

PM-RAIRS measurements were carried on a Nicolet Nexus 5700 FT-IR spectrometer (Madison, WI, USA) equipped with a wide band HgCdTe detector cooled with liquid nitrogen. Infrared spectra were obtained by coaddition of 128 scans at 8 cm^−1^ resolution,. A ZnSe photoelastic modulator and a ZnSe grid polarizer were placed prior to the sample in order to modulate the incident beam between p and s polarizations (PM90), modulation frequency = 36 kHz HINDS Instruments Inc., (Hillsboro, OR, USA). Interferograms (sum and difference) were processed via Fourier-transformation to obtain the resulting PM-RAIRS signal, which is the differential reflectivity (ΔR/R°) = (Rp − Rs)/(Rp + Rs), with Rs and Rp being the signals perpendicalur and parallel to the incident plane, respectively.

### 3.5. X-Ray Photoelectron Spectroscopy (XPS)

XPS analyses were performed using an Omicron Argus spectrometer (Taunusstein, Germany) equipped with a monochromated AlK_α_ radiation source (*h*ν = 1486.6 eV) working at an electron beam power of 300 W. Photoelectrons emission was analyzed at a takeoff angle of 90°; the analyses were carried out under ultra-high vacuum conditions (≤10^−10^ Torr) after introduction via a loadlock into the main chamber. Spectra were obtained by setting up a 100 eV pass energy for the survey spectrum and a pass energyof 20 eV was chosen for the high resolutions regions. Binding energies were calibrated against the Au4f_7/2_ binding energy at 84.0 eV. Element peak intensities were corrected by Scofield factors [47]. Casa XPS v.2.3.15 software (Casa Software Ldt, Teignmouth, UK) was utilized to fit the spectra and Gaussian/Lorentzian ration was applied (G/L ration = 70/30).

### 3.6. Scanning Electron Microscopy with Field Emission Gun (SEM-FEG)

SEM images were recorded with a SU-70 field emission gun scanning electron microscope (Hitachi, Tokyo, Japan). The samples (fixed on SEM support with a carbon adhesive tape) were observed without metallization. An in-lens secondary electron detector (SE_Upper_) was used to record SEM images of surfaces. The accelerating voltage was 1 kVwith a working distance of 5 mm. Few different locations were analyzed on each surface to obtain a statistical view of the entire surface; thus, at least 100 single bacteria were observed.

### 3.7. Water Contact Angle Measurements

Static water contact angles (DSA100 apparatus, Krüss, Hamburg, Germany) were measured under ambient conditions (at 20 °C and 40% relative humidity) by analyzing the profile of sessile drops (1-μL droplet of milliQ water) deposited on a given surface. The drop profile was recorded by a CCD camera, while the angle were measured by the image analysis software. For each analyzed surface, at least for different location were chosen for the deposition of the droplet; each test was performed in triplicate on three different samples. The reported values for a given surface are thus the averages of these 12 measurements.

### 3.8. Bacterial Strain and Culture Conditions

Non-pathogenic bacteria *Listeria ivanovii* Li4pVS2 (Courtesy of Prof. J.-M. Berjeaud, Poitiers University, Poitiers, France) were used to investigate the antibacterial activity of the modified surfaces. Bacterial suspensions were prepared from frozen cultures incubated overnight (37 °C under agitation at 250 rpm) in brain heart infusion (BHI) broth (BD Difco, Franklin Lakes, NJ, USA). After centrifugation at 10,000× *g* for 5 min and elimination of the liquid phase (supernatant) the bacteria were redisposed in NaCl 0.9% solution up to a concentration of to 2 × 10^6^ colony forming unity (CFU) per mL, verified by measuring the OD_620nm_ = 0.02.

### 3.9. Deposition of Bacteria on Samples

Prior to bacteria deposition, surfaces were rinsed in a 70% ethanol aqueous solution and dried in sterile environment. Each sample was deposited in a 12-well plate containing 2 mL of bacteria inoculum freshly prepared. All surfaces were in contact with the bacterial inoculum for 3 h at room temperature under a relative wet atmosphere, unless otherwise stated.

### 3.10. Evaluation of Bacteria Adhesion by Infrared Spectroscopy

After contact with bacterial solutions, surfaces were washed five times with 2 mL batch of isotonic sterile solution (NaCl 0.9%) and dried under sterile flow. For each surface, and in order to scan their entire surface and collect the signal from all the adhered bacteria, 10 infrared spectra were acquired by PM-RAIRS. All measurements (control and functionalized surfaces) were carried out during the same experimental session, to reduce set-up variations such as beam intensity of background variation. The relative amounts of bacteria adsorbed were evaluated by considering the amide bands area, the bacteria IR fingerprints. It is important to note that the temporin SAMs already have a amide I and II signature, thus the area due to the peptides have been subtracted from the ones obtained on SAMs + bacteria surfaces in order to obtain the area due only to bacteria. The areas were integrated from 1700 to 1500 cm^−1^ to include the whole signal due to amides I and II contributions, respectively, at 1660 and 1550 cm^−1^. A normalization upon bare gold surface was done using the following adhesion equation: Ad (%) = 100 × (area of amide bands on sample)/(area of amide bands on gold). The results are presented with uncertainty coming from the propagation of the uncertainties attached to the IR measurement. These results were confirmed by repeating the same procedure in triplicates.

### 3.11. Observation of Bacterial Morphology by Microscopy

Bacterial morphology analyses were carried out by SEM-FEG imaging, in order to visualize the potential effect of functionalized surfaces upon contact with bacteria. Since SEM-FEG observations is a high vacuum technique, surfaces were prepared by fixing bacterial state using glutaraldehyde reticulation. Thus, surfaces were immersed 2 h in 2.5% glutaraldehyde (*v*/*v* in PBS) solution. After PBS rinsing, surfaces were gently dried using ethanol solutions of increasing concentrations (25, 50, 75, 96 and 100%) before being dried.

### 3.12. Bacteria Growth Capacity

Bacterial growth capacity after contact with functionalized surfaces was done as follows. Gold substrates functionalized and bare gold substrates were set up separately in a well of 12-wells plates; 2 mL of bacterial suspension in NaCl 0.9% baring a DO at 620 nm = 0.02, leading to a bacterial concentration of 2 × 10^6^ CFU/mL, were filled in each well. After bacterial deposition during 3 h at room temperature, the surfaces were washed five times with 2 mL batch of saline solution NaCl at 0.9%, in order to remove non-adhered bacteria. Surfaces were then transferred into a sterile tube containing 2 mL of saline sterile solution and sonicated (Bandelin Sonorex RK 31, Berlin, Germany; f = 35 kHz, P = 90 W) during 5 min to recover most of the adhered bacteria without damaging them. SEM-FEG images where recorded post sonication ensuring that most of adhered bacteria were detached during the sonication process. The recovered bacteria suspensions were diluted 100 and 1000 times before deposition of 20 μL of each dilution in triplicate on BHI-agar plates. The plates were incubated at 37 °C overnight before enumeration. Results were expressed in percentages of the number of attached and cultivable bacterial cells onto the different surfaces, as compared to gold substrates: %growth = 100 × (number of CFU on sample)/(number of CFU on bare gold substrate). The results were also expressed as %killing, where %killing = 100 − %growth. These tests were done in triplicate for each surface and the percentage of growth (killing) was averaged over three given surfaces. In order to obtain %killing corrected by the adhesion factor, the raw CFU data were normalized toward the 100% adhesion on bare gold surfaces. Thus, % killing were again calculated with the same above equation to obtain the corrected values of growth/killing.

### 3.13. Minimal Inhibitory Concentration (MIC) and Minimal Bactericidal Concentration (MBC) Determination

MIC values of temporin analogs were determined using a liquid growth inhibition assay. An exponential-phase bacterial culture of *Listeria ivanovii* was diluted in BHI broth to an OD of 0.02 (2 × 10^6^ CFU/mL). A volume of 100 μL of this bacterial suspension was mixed with 100 μL of 2-fold serial aqueous dilutions of each temporin (final concentrations from 0.78 to 100 µM). The bacterial growth was monitored by measuring the OD change during overnight incubation. MIC and MBC tests were carried out in triplicate with positive (0.7% formaldehyde) and negative (saline NaCl 0.9% solution) controls. MIC is expressed as the lowest concentration that completely inhibits bacterial growth after overnight incubation. The bacterial suspension corresponding to the MIC well and 4 wells above MIC were collected and deposited on agar plate in order to determine the minimal bactericidal concentration (MBC). The MBC is expressed as the lowest concentration for which a decrease of at least 99.99% is observed on agar plates after overnight incubation.

## 4. Conclusions

In this study we have shown the successful covalent grafting of analogs of temporin-SHa antimicrobial peptide on gold surfaces. The modifications of the analogs together with the anchoring mode of the peptide, randomly via the amine groups or oriented via the C-ter carboxyl group, do not influence the surface density, and each surface is homogeneously covered with around 1 peptide/nm². Antimicrobial activity was demonstrated for free temporins in solution towards the gram-positive bacterial strain *L. ivanovii*, and grafted active peptides retained also this antimicrobial activity. Although the anchoring mode of the peptide does not influence bacterial activity, the use of a non-natural all-D configuration peptide, D-[K^3^]SHa, increases by approximately 15% this antibacterial activity. Interestingly, in addition to its higher activity, this D-enantiomer has shown to reduce by 50% the bacterial adhesion on modified gold surfaces. Regarding the engineering of biofouling surfaces, it is clear that the antiadhesive character goes hand in hand with the killing efficiency, and the use of all-D molecules will certainly open a wide range of possibilities when considering one-pot antiadhesive/antibacterial modified surfaces. Finally, one should keep in mind that this study represents a proof of concept on the possible tuning between antiadhesive and bactericidal properties towards a single Gram positive bacterial strain, and early successful experiments on other strains (Gram negative and Gram positive) are revealing all the potential of temporin analogs surface functionalization.

## Figures and Tables

**Table 1:** Anti-*Listeria* activity of temporin analogs in solution and different peptides characteristics. MIC: minimal inhibitory concentration. MBC: minimal bactericidal concentration. Lowercase letters indicate d-amino acid residues.

Temporin	MIC (µM)	MBC (µM)	Sequence	Net Charge	Mw ^a^	GRAVY ^b^
**SHa**	6	6	FLSGIVGMLGKLF_amide_	+2	1380.76	1.67
**[A^2,6,9^]SHa**	>200	>200	FASGIAGMAGKLF_amide_	+2	1268.55	1.18
**[K^3^]SHa**	1.56	3.12	FLKGIVGMLGKLF_amide_	+3	1421.86	1.43
**d-[K^3^]SHa**	1.56	3.12	flkGivGmlGklf_amide_	+3	1421.86	1.43
**[A^2,6,9^, K^3^]SHa**	>200	>200	FAKGIAGMAGKLF_amide_	+3	1309.64	0.94
**[K^3^]SHa-COOH**	64	128	FLKGIVGMLGKLF_COOH_	+3	1422.86	1.43
**[A^2,6,9^, K^3^]SHa-COOH**	128	256	FAKGIAGMAGKLF_COOH_	+3	1310.64	0.94

^a^ Average molecular mass (http://rna.rega.kuleuven.be/masspec/pepcalc.htm). ^b^ Grand average of hydropathicity (https://web.expasy.org/protparam/).

**Table 2:** Atomic percentage composition of functionalized surfaces obtained from XPS data presented in Figure 3.

	C1s	O1s	N1s	S2p
**MUAM**	79.2	13.1	4.3	3.4
**MUAM-[K^3^]SHa-COOH**	76.4	15.4	6.8	1.4
**MUA**	75.0	21.0	-	4.0
**MUA-[K^3^]SHa**	72.9	17.7	6.7	2.7

**Table 3:** Peptide and surface physicochemical properties. The molecular weight and the net charge of unbound peptides are indicated. The estimated thickness and molecular surface density of the thiol and thiol-temporin layers were calculated using XPS data.

	MUA	[K^3^]SHa	d-[K^3^]SHa	[A^2,6,9^, K^3^]SHa	MUAM	[K^3^]SHa-COOH	[A^2,6,9^, K^3^]SHa-COOH
**Molecular weight (Da)**	218.4	1421.9	1421.9	1309.6	239.8	1422.9	1310.6
**Net charge**	N/A	+3	+3	+3	N/A	+2	+2
**Total thickness (Å)**	10.6 ± 2.0	32.0 ± 2.9	29.2 ± 4.8	27.6 ± 3.5	11.4 ± 2.0	28.1 ± 2.1	26.9 ± 2.0
**Component thickness (Å)**	10.6 ± 2.0	21.4 ± 2.6	18.6 ± 2.7	17.0 ± 2.6	11.4 ± 2.0	16.7 ± 2.4	15.5 ± 2.4
**Molecular surface density** **(×10^14^ molecules/cm²)**	3.2 ± 0.6	1.0 ± 0.1	0.9 ± 0.1	0.85 ± 0.1	3.1 ± 0.5	0.8 ± 0.1	0.8 ± 0.1

**Table 4:** Bacterial killing, water contact angle (WCA) and bacterial adhesion of the different modified surfaces (SAM with and without grafted temporin) compared to bare gold surface. Raw Killing (killing without adhesion correction) and corrected killing (killing with adhesion correction) are both indicated.

	Au	MUA	[K^3^]SHa	D-[K^3^]SHa	[A^2,6,9^, K^3^]SHa	MUAM	[K^3^]SHa-COOH	[A^2,6,9^, K^3^]SHa-COOH
**Raw Killing (%)**	N/A	13.5 ± 0.2	78.9 ± 0.6	86.4 ± 1.7	7.6 ± 0.9	9.1 ± 5.3	78.8 ± 2.8	22.1 ± 2.2
**WCA (°)**	78 ± 2	56 ± 3	72 ± 3	65 ± 2	67 ± 3	82 ± 1	73 ± 2	70 ± 2
**Adhesion (%)**	100	114 ± 15	124 ± 25	66 ± 15	104 ± 22	130 ± 25	105 ± 10	122 ± 18
**Corrected Killing (%)**	N/A	1.4 ± 0.2	77.0 ± 0.7	92.1 ± 1.0	3.8 ± 0.5	3.2 ± 1.8	78.3 ± 2.6	4.9 ± 0.7
